# Safety and Feasibility of the RevCore Catheter for Venous In-Stent Thrombosis: A Multicenter, Retrospective Study

**DOI:** 10.1016/j.jscai.2025.102571

**Published:** 2025-03-20

**Authors:** Abdullah Shaikh, Angelo G. Marino, Michael Siah, Min H. Choi, Steven D. Abramowitz

**Affiliations:** a Department of Radiology, Allegheny Health Network Research Institute, Pittsburgh, Pennsylvania; b Department of Radiology & Biomedical Imaging, Yale School of Medicine, New Haven, Connecticut; c Department of Vascular Surgery, UT Southwestern Medical Center, Dallas, Texas; d Department of Vascular Surgery, MedStar Health, Washington, DC

**Keywords:** deep-vein thrombosis, in-stent thrombosis, mechanical thrombectomy, venous stents

## Abstract

**Background:**

After venous stent placement, patients may develop in-stent thrombosis (IST). The RevCore thrombectomy catheter (Inari Medical) is a novel device specifically designed to treat venous IST. Herein, the safety and feasibility of this device are evaluated.

**Methods:**

Patients were retrospectively included if they were ≥18 years old, had lower extremity venous IST, and were treated using the study device. The primary end point is technical success, defined as an average postprocedural effective diameter ≥50% and calculated using stent diameter and flow channel diameter on intravascular ultrasound. The safety end points are 30-day device-related serious adverse events, namely mortality, readmission, clinically significant pulmonary embolism, and vessel perforation.

**Results:**

Data from 44 patients (48 treated limbs, 6 treated stent types) from 4 centers were analyzed. Mean age was 54.8 ± 17.1 years, and 23 (52.3%) patients were female. Median IST symptom duration was 8.0 (IQR, 2.0-104.0) weeks. The primary end point was achieved in all 48 limbs. Comparing baseline with postprocedural measurements, effective stent diameter increased from 21.1% ± 26.5% to 89.6% ± 10.1%. No patient met a safety end point. On the first follow-up at 40.0 (29.0-62.5) days, primary patency was demonstrated in 32 (94.1%) of the 34 treated limbs with available data. Six (85.7%) limbs with Clinical-Etiology-Anatomy-Pathophysiology scores of C_6_ at baseline had longer-term data available (3.1 ± 0.7 months), and all limbs demonstrated improvement of C_6_ disease, with 3 (50.0%) improving to class C_5_.

**Conclusions:**

A thrombectomy procedure for deep-vein IST reestablished patency in all cases and across multiple stent types. Patency was sustained through the first follow-up in 94% of limbs.

## Introduction

Patients with central or proximal lower extremity venous obstructions may experience claudication, leg swelling, and skin changes, including venous ulceration in severe cases.^1^ Venous obstructions can be thrombotic, including deep-vein thrombosis (DVT) and associated postthrombotic material, or can be nonthrombotic, such as May-Thurner Syndrome in which patients demonstrate compressive iliac lesions.^2^ Stent placement is an important component of effective treatment for many patients with these types of venous disease.^1^^,^^3^, ^4^, ^5^, ^6^ However, after placement of a venous stent, subsequent in-stent thrombosis (IST) can occur in up to 25% of patients within 2 to 3 years and cause symptom recurrence.^7^^,^^8^ Patients with postthrombotic disease are at greatest risk for IST.^8^, ^9^, ^10^

Cases of IST are often challenging to treat, and available therapies are relatively limited.^11^ Venoplasty and stent relining are standard approaches for intervening in IST, but these strategies are frequently ineffective for reestablishing and maintaining stent patency.^12^^,^^13^ Fibrinolytic-based therapies are also commonly used; however, the presence of organized postthrombotic material can lead to treatment failure, and the risk of major bleeding may be disproportionately high.^14^ As a result, many patients with IST are not treated or receive insufficient care. New technologies are needed to address this population more effectively.

A device with the potential to improve outcomes for patients with venous IST has recently become available. The RevCore thrombectomy catheter (Inari Medical), which has a reinforced shaft and an expandable element for the removal of both acute and nonacute thrombus, is designed for the percutaneous treatment of IST. Several case reports describe successful outcomes when using the device in this context.^15^, ^16^, ^17^ However, quantitative data regarding the feasibility and safety of the device have not yet been reported. This is the first multiinstitutional study to present outcomes for patients with venous IST who were treated using this unique mechanical thrombectomy device.

## Methods

### Study design

This multicenter retrospective analysis includes all patients who were: (1) ≥18 years old; (2) diagnosed with symptomatic IST of the inferior vena cava (IVC), common iliac vein, external iliac vein, or common femoral vein, either alone or in combination; and (3) treated using the study device at a participating hospital. To receive thrombectomy treatment, the stent was required to be in place >6 weeks, ≥10 mm in diameter, and free of struts that were broken or protruding into the stent lumen. There were no other eligibility criteria.

Patient data were deidentified before the analysis occurred. Thus, institutional review board approvals were waived. This study followed the principles outlined in the Declaration of Helsinki.

### Procedural details

#### Study device

**Figure 1:**
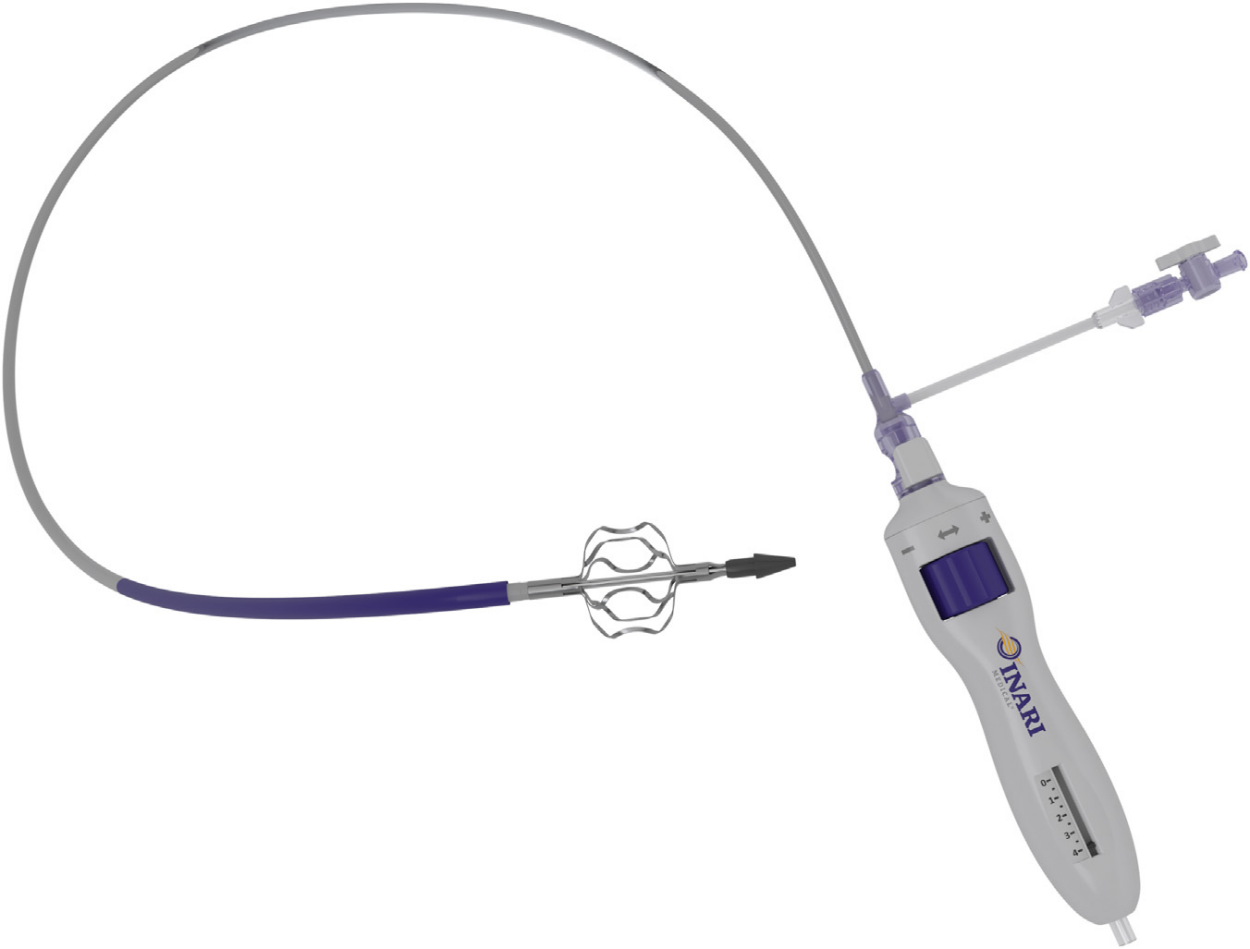
**The RevCore thrombectomy catheter (Inari Medical).** The study device is designed to treat in-stent thrombosis and includes a coring element at the distal tip. A dial on the catheter handle can be adjusted to expand or collapse the element to the desired size. Here, the element is shown fully expanded to a diameter of 20 mm.

In all cases, IST was treated with mechanical thrombectomy using the study device, the RevCore catheter (Figure 1). The device is an over-the-wire catheter indicated for the removal of acute-to-chronic thrombus, including within obstructed venous stents, by maneuvering an expandable element located at the distal end of the system. The element is designed to avoid entanglement in the struts and cells of a stent. The device does not include a built-in means of embolic protection, and, therefore, simultaneous use of a proximal device to capture possible mobilized thrombus is recommended.

#### Prethrombectomy steps

Before IST thrombectomy, all patients underwent physical examination and imaging studies via computed tomography venography or duplex ultrasound to confirm the diagnosis of IST. Patients were placed under moderate or general anesthesia and received systemic anticoagulation with heparin to maintain an activated clotting time of 250 to 300 seconds throughout the procedure. Ultrasound guidance was used to obtain venous access. In many cases, the right internal jugular vein was accessed for delivery of the study device, for through-and-through wire delivery between a lower extremity access site, and/or for placement of a device in the IVC to entrap potential procedural emboli. Intravascular ultrasound was used before thrombectomy with the study device for the following reasons: (1) to confirm that a preplaced 0.035" Amplatz Super Stiff guide wire (Boston Scientific) was not placed through a stent cell and that the treatment area within the stent was ≥10 mm, (2) to assess stent integrity and degree of obstruction at baseline, and (3) to evaluate adjacent segments for disease.

#### Mechanical thrombectomy with the study device

**Figure 2:**
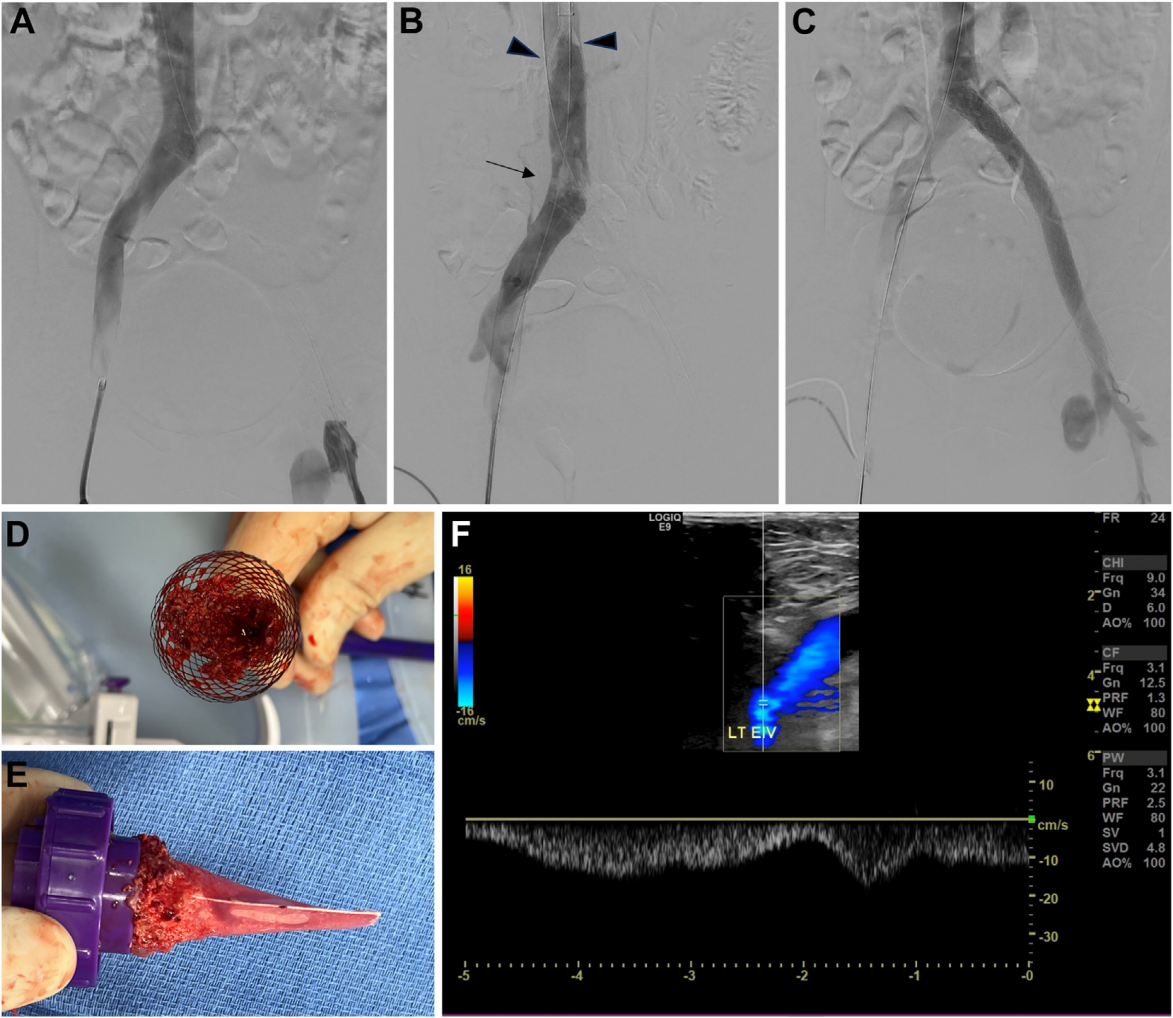
**Example images from a case of in-stent thrombosis (IST) treated using the study device.** (A) Prethrombectomy venogram with simultaneous contrast injections from bilateral popliteal vein access sites revealing occlusive IST of a previously relined stent in the left common and external iliac veins (right side). (B) Venogram acquired after multiple passes with the thrombectomy catheter demonstrating liberated thromboemboli both in the Protrieve sheath (Inari Medical) funnel in the inferior vena cava (arrowheads) and adherent to the proximal crown of the stent (arrow). (C) Postthrombectomy completion venogram showing restoration of complete stent patency. Emboli were removed throughout the procedure via Protrieve aspiration, with some remaining in the mesh funnel upon removal (D). (E) Aspirated blood was filtered and returned to the patient, and emboli were also observed in the filter of the FlowSaver (Inari Medical) blood return system. (F) Duplex ultrasound performed during follow-up at approximately 2 weeks demonstrated maintenance of complete patency and flow.

When appropriate, venoplasty was first used to create a lumen within the thrombosed segment. The handle of the study device includes a meter with a range of 0 to 4 to indicate the degree of element expansion; with the thrombectomy element fully contracted in the “0” position and covered by the delivery catheter, the device was inserted and advanced under fluoroscopic visualization until the tip of the catheter was beyond the thrombosis. Next, the element was uncovered but left contracted during a first retraction at 1 to 2 mm per second while rotating, or “revving,” the catheter handle. After retracting and revving through the entire thrombosed segment, back-and-forth scrubbing along the stent was performed, with particular care taken when scrubbing regions of a stent crown or overlap. The catheter was then readvanced, and the element was expanded to a setting of 0.5. To continue removing obstructive material, the element was again retracted while revving the device handle and then used for scrubbing. This process was repeated until fluoroscopy and tactile feedback indicated achievement of complete stent patency or further passes yielded no additional luminal gain. Adjunctive venoplasty was performed within the stent as needed. Any embolized thrombus was removed by aspiration thrombectomy. Figure 2 presents images from an example case.

#### Postthrombectomy steps

After thrombectomy, final imaging with venography and intravascular ultrasound was performed. All devices and sheaths were removed from the patient. Hemostasis was achieved at all access sites using sutures or manual compression. Most patients were discharged home on the same day, but some patients stayed in the hospital if needed. All patients were discharged home on anticoagulation and antiplatelet regimens; patients were prescribed clopidogrel and either enoxaparin, rivaroxaban, or apixaban for 2 to 4 weeks and were then transitioned to aspirin and either rivaroxaban or apixaban. Patients were advised to wear knee-high compression therapy if edema in the lower extremities was present. Follow-up imaging was performed by computed tomography venography or ultrasound per institution protocol. A follow-up clinic visit was also scheduled.

### Data collection: End points and additional outcomes

The primary end point of the study is technical success, defined as achieving an effective diameter of ≥50% at procedure completion when assessed by intravascular ultrasound. Effective diameter is calculated as the average patent lumen diameter divided by the stent diameter. Complete or near-complete stent patency is defined as an effective diameter ≥75%, and partial stent patency is considered an effective diameter <75% without occlusion. For patients with multiple treated stents, data for the stent with the least patency was used in the analysis of limb-level variables at all time points. The safety end points are 30-day device-related serious adverse events (SAE), encompassing mortality, readmission, vessel perforation, and clinically significant pulmonary embolism (PE). Mortalities and readmissions were classified as device related if a preceding adverse event was judged to have been caused by the device. Clinically significant PE is defined as systemic hypotension or elevated troponin with right ventricular dysfunction demonstrated by echocardiography or computed tomography angiography.^18^

Additional collected data include demographics, medical history, clinical presentation details, and procedural characteristics, such as luminal gain and the need for stent relining. Symptom duration is categorized as acute, subacute, or chronic, defined as ≤2 weeks, >2 weeks but <4 weeks, and ≥4 weeks, respectively. The age of extracted thrombus is also evaluated by postthrombectomy visual and tactile inspection using thrombus chronicity definitions established in previously published literature describing mechanical thrombectomy outcomes for the treatment of DVT, with acute thrombus defined as dark red and gelatinous, subacute thrombus as lighter red and more organized, and chronic thrombus as pink and rubbery.^19^^,^^20^ Follow-up measurements include device-related rethrombosis, defined as IST recurrence after an adverse event that was determined to have been related to the device; reinterventions; and clinical improvements in Villalta score, edema, and pain. Physician-assessed edema and patient-reported pain were collected as absent, mild, moderate, or severe.

### Data analysis

Data collection and analysis were performed using Excel (Microsoft). Results are presented as mean ± SD, median (IQR), or counts (%). Statistical comparisons were performed using Wilcoxon signed-rank tests for continuous variables and McNemar-Bowker tests for categorical variables. A *P* value <.05 was considered statistically significant. Kaplan-Meier curves were created using R v4.4.0 (R Foundation for Statistical Computing).

## Results

### Baseline characteristics and clinical presentation

The analysis included 44 patients with lower extremity and/or central IST who were treated at 4 centers between March and November 2023. In total, 65 stents in 48 limbs underwent mechanical thrombectomy with the study device. Mean age was 54.8 ± 17.1 years, and 23 (52.3%) patients were female (Table 1). Four (9.1%) patients had bilateral IST. Median symptom duration was 8.0 (2.0-104.0) weeks, with 11 (24.4%) limbs having acute symptoms, 5 (11.1%) limbs having subacute symptoms, and 29 (64.4%) limbs having chronic symptoms. There was a history of DVT in 46 (95.8%) limbs, with only 2 having originally received the stent to address a nonthrombotic iliac vein lesion. Eleven (25.0%) patients had undergone a prior intervention for the index IST, with treatments including venoplasty, catheter-directed thrombolysis, stent relining, or another form of mechanical thrombectomy; 1 of these 11 patients had acute IST symptoms, and the rest had chronic symptoms.

**Table 1:** Baseline characteristics and clinical presentation.

Characteristic	Patient (N = 44)
Age, y	54.8 ± 17.1
Female sex	23 (52.3)
Body mass index, kg/m^2^	30.6 ± 5.7
Bilateral IST	4 (9.1)
Prior anticoagulation	30 (69.8)
Prior intervention^a^	11 (25.0)
Venoplasty	7 (63.6)
Fibrinolytics	5 (45.5)
Stent relining	6 (54.5)
Other mechanical thrombectomy	5 (45.5)
Treated limbs	N = 48
Symptom duration, wk	8.0 (2.0-104.0)
Unprovoked	21 (60.0)
Villalta score	9.0 ± 5.1
CEAP clinical class	
C_0_	1 (2.1)
C_1_	1 (2.1)
C_2_	0 (0)
C_3_	22 (46.8)
C_4_	13 (27.7)
C_5_	3 (6.4)
C_6_	7 (14.9)
Any edema	43 (93.5)
Any pain	37 (84.1)
Patency	28 (43.1)
Complete or near complete^b^	0 (0)
Partial^c^	21 (43.8)
Occluded	27 (56.3)
Average effective diameter, %	21.1 ± 26.5
Average flow channel diameter, mm	3.0 ± 3.8
Average flow channel area, mm^2^	16.3 ± 26.8
Treated stents	N = 65
Wallstent (Boston Scientific)	31 (53.4)
Abre (Medtronic)	13 (22.4)
Venovo (BD Medical)	11 (19.0)
E-Luminexx (BD Medical)	1 (1.7)
Vici (Boston Scientific)	1 (1.7)
Zilver Vena (Cook Medical)	1 (1.7)

Data presented as mean ± SD, n (%), or median (IQR).

CEAP, Clinical-Etiology-Anatomy-Pathophysiology; IST, in-stent thrombosis.

a Some patients received multiple forms of prior treatment for the index IST.

b Patency ≥75%.

c Patency <75% without occlusion.

**Figure 3:**
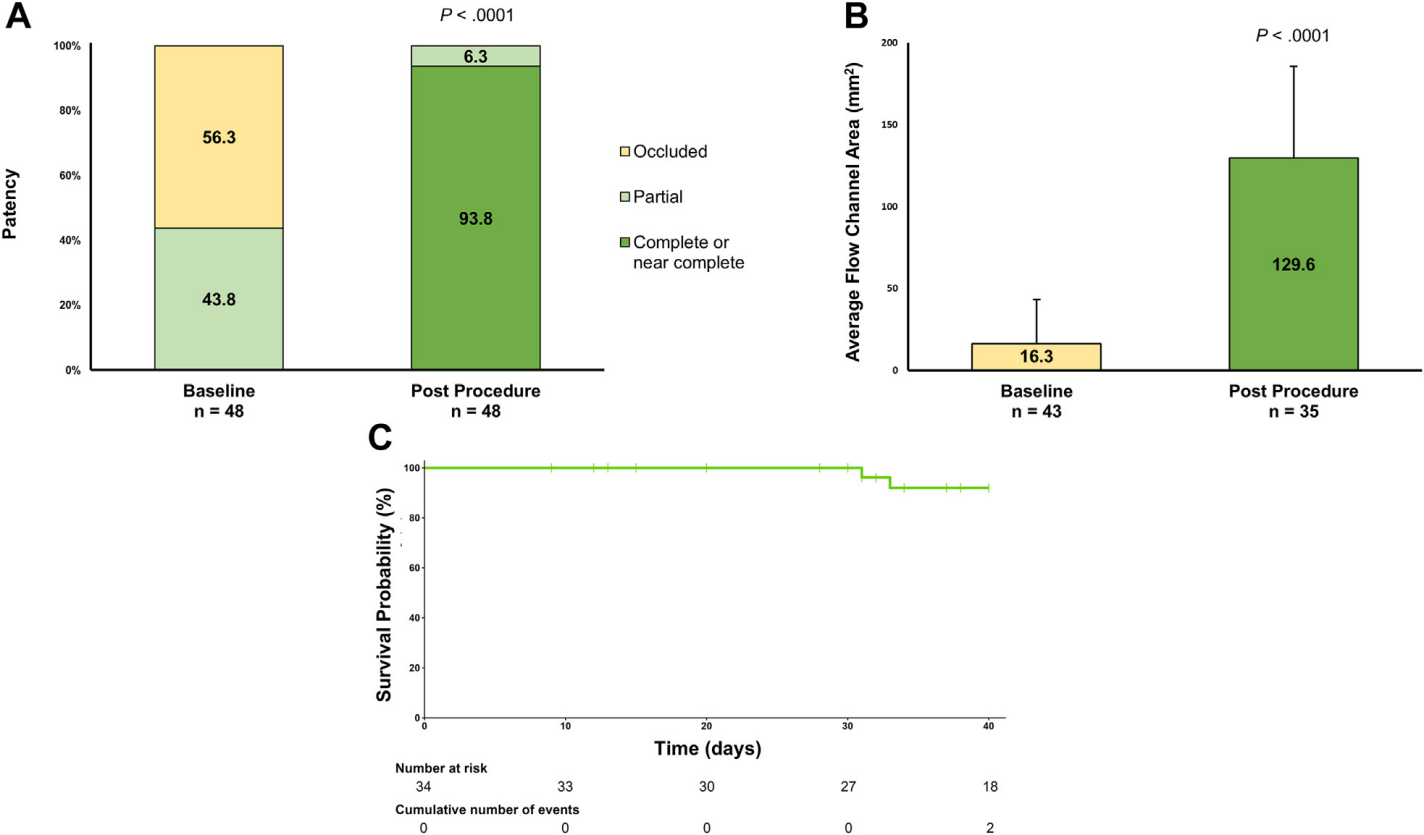
**Patency outcomes.** (A) Limb-level stent patency at baseline and immediately after the procedure. (B) Average flow channel area at baseline and immediately after the procedure, presented as means with SD. (C) Limb-level stent patency at first follow-up plotted through the median time to first follow-up of 40 days; all stent occlusion events through first follow-up occurred within 40 days of the procedure.

The mean Villalta score was 9.0 ± 5.1. Seven (14.9%) limbs demonstrated C_6_ disease by Clinical-Etiology-Anatomy-Pathophysiology (CEAP) classification. Occlusion was observed in 27 (56.3%) limbs, and all remaining limbs demonstrated partial patency (Figure 3A). The study included a variety of treated stent types, with the Wallstent (Boston Scientific) being the most common (31 [53.4%]).

### Procedural and discharge characteristics

Forty-six procedures were performed. One patient with bilateral IST received treatment for the contralateral leg in a planned second procedure, and another patient developed IST in the contralateral limb after successful treatment of the first leg. Thus, interventions were single session for 43 (97.7%) patients. In 16 (34.8%) procedures, the study device was deployed exclusively via right internal jugular vein access (Table 2). A mean of 4.7 ± 1.9 passes with the study device were performed. A secondary device was used to trap potential procedural emboli in all (100%) cases, with the Protrieve sheath (Inari Medical) used for embolic capture in 43 (93.5%) procedures. Procedural embolus was captured in 36 (83.7%) cases. The median estimated thrombus volume removed from target segments based on intravascular ultrasound imaging and venograms acquired at the beginning and end of the procedure was 90.0% (60.0%-90.0%), with the obstructive material appearing at least partly chronic on postthrombectomy inspection in 28 (84.8%) cases. The study device time was 28.8 ± 13.5 minutes, and the median estimated blood loss was <30 mL. Adjunctive lytic agents were given to a single (2.2%) patient. One (1.5%) stent fracture was noted after a procedure involving a patient with chronic IST symptoms and a partially relined stent in which the proximal end of the newer stent was not apposed to the wall of the original stent. In this case, crossing proved challenging, and the experienced operator considered the cause of the fracture to be the adjunctive balloon venoplasty performed before thrombectomy. There were no (0%) instances of stent migration or thrombectomy element entanglement in a stent. In 4 (8.7%) procedures requiring relining for persistent IST, 60.0% ± 26.5% of thrombus was removed, and thrombus was classified as chronic in each of the 4 cases. All new stents were placed during the thrombectomy procedure.

**Table 2:** Procedural and discharge characteristics.

Characteristic	Procedure (N = 46)
Diagnostic IVUS use	46 (100)
Study device used exclusively via right internal jugular vein	16 (34.8)
Device passes	4.7 ± 1.9
Embolic capture device use	46 (100)
Procedural embolus captured	36 (83.7)
Stent thrombus removal, %	90.0 (60.0-90.0)
Thrombus chronicity on postthrombectomy inspection^a^	
Chronic	28 (84.8)
Subacute	3 (9.1)
Acute	1 (3.0)
Thrombectomy time, minutes	28.8 ± 13.5
Adjunctive therapies	
Prethrombectomy fibrinolytics	1 (2.2)
Postthrombectomy fibrinolytics	0 (0)
Prethrombectomy venoplasty	18 (39.1)
Postthrombectomy venoplasty	40 (87.0)
New stent placed	17 (37.0)
Stent relined for residual thrombosis	4 (8.7)
Postprocedural hospital stay	19 (45.2)
Postprocedural hospital LoS, d	0 (0-2)
Postprocedural ICU stay	1 (2.4)
Treated limbs	N = 48
Immediate postprocedural effective diameter ≥ 50%	48 (100)
Immediate postprocedural average effective diameter, %	89.6 ± 10.1
Immediate postprocedural average flow channel diameter, mm	13.2 ± 2.3
Immediate postprocedural average flow channel area, mm^2^	129.6 ± 55.9
Immediate postprocedural patency	48 (100)
Complete or near complete^b^	45 (93.8)
Partial^c^	3 (6.3)
Occluded	0 (0)
Treated stents	N = 65
Stent migration or entanglement	0 (0)
New stent fracture	1 (1.5)

Data presented as n (%), mean ± SD, or median (IQR).

ICU, intensive care unit; IST, in-stent thrombosis; IVUS, intravascular ultrasound; LoS, length of stay.

a Based on most chronic thrombus present.

b Patency ≥75%.

c Patency <75% without occlusion.

At procedure completion, all 48 (100%) limbs met the primary end point of effective diameter ≥50%. Forty-five (93.8%) limbs demonstrated complete or near-complete stent patency (Figure 3A). Comparing baseline with postprocedural measurements, the effective diameter increased from 21.1% ± 26.5% to 89.6% ± 10.1% (*P* < .0001), a 4.2× improvement, and the average flow channel area increased from a mean of 16.3 ± 26.8 mm^2^ to 129.6 ± 55.9 mm^2^, (*P* < .0001, Figure 3B), an 8.0× improvement.

A hospital stay was not required after 23 (54.8%) procedures. The median postprocedural hospital length of stay was 0 (0-2) days, and an intensive care unit stay was required after 1 (2.4%) procedure for intraprocedural cardiac arrest.

### Safety, patency, and clinical outcomes at follow-up

From available data, including results for 39 procedures on 37 patients, the time to first follow-up was 40.0 (29.0-62.5) days (Table 3). None of the patients met a safety end point, as there were no device-related SAE at 30 days, including 0% mortality, readmission, PE, and vessel perforation. In addition to the absence of PE meeting the safety end point definition, no PE events were demonstrated during the procedure or during the follow-up period.

**Table 3:** Safety, patency, and clinical outcomes at follow-up.

Outcome	Procedure (N = 39)
Time to follow-up, d	40.0 (29.0-62.5)
Safety outcomes	
30-day device-related SAE	0 (0)
Mortality	0 (0)
Readmission	0 (0)
Pulmonary embolism	0 (0)
Vessel perforation	0 (0)
Clinical outcomes	Limb (N = 39)
Villalta score	5.6 ± 4.8
CEAP clinical class	
C_0_	1 (2.8)
C_1_	6 (16.7)
C_2_	2 (5.6)
C_3_	12 (33.3)
C_4_	8 (22.2)
C_5_	4 (11.1)
C_6_	3 (8.3)
Edema improvement or maintained absence	14 (48.3)
Pain improvement or maintained absence	17 (63.0)
Primary patency	32 (94.1)

Data presented as mean ± SD or n (%).

CEAP, Clinical-Etiology-Anatomy-Pathophysiology; SAE, serious adverse event.

Four patients experienced adverse events relevant to the procedure. One patient developed an access site infection requiring hospital admission for intravenous antibiotic treatment. Another patient experienced an intraprocedural cardiac arrest that necessitated extracorporeal membrane oxygenation, but venography showed no evidence of PE or occlusive IVC thrombus; the patient recovered, but a pseudoaneurysm at 1 of the extracorporeal membrane oxygenation sites required repair. A third patient with a history of lupus, iron deficiency anemia, iron infusions, and heavy menstruation exhibited a postprocedural hemoglobin measurement of 5.5 g/dL despite an estimated blood loss of 20 mL during the procedure, and this patient required a transfusion of 2 units of packed red blood cells. The final patient, who had weakness at baseline and was concurrently undergoing assessment for orthostatic hypotension, reported weakness of the treated leg during the 1-day postprocedural hospital stay, but magnetic resonance imaging was negative for acute pathology and revealed degenerative disc disease.

Of the 39 limbs assessed at a follow-up visit, 34 of the limbs had stent patency data available, and 32 (94.1%) of these limbs demonstrated primary patency. Two (5.9%) limbs showed stent occlusion at the first follow-up, with both limbs losing patency prior to the median time to the first follow-up (Figure 3C) and having demonstrated chronic thrombus on postthrombectomy inspection. One occlusion led to reintervention with the study device, and the second occlusion occurred in a patient who was noncompliant with their anticoagulation regimen and developed a new pelvic mass surrounding the stent. Villalta score at the first follow-up was 5.6 ± 4.8, an improvement of –3.4 compared with the baseline.

Longer-term data were available for 21 limbs. Rethrombosis necessitating reintervention occurred after the first follow-up in 2 (9.5%) limbs, one at approximately 3 months after thrombectomy and the other after roughly 6.5 months. Patency was successfully restored in both stents using the study device, yielding a secondary patency of 100%. Six (85.7%) of the limbs with a baseline CEAP classification of C_6_ had follow-up data available from 3.1 ± 0.7 months after mechanical thrombectomy. All (100%) of these limbs showed improvement of C_6_ venous disease as assessed by wound area, with paired measurements demonstrating a decrease from 9.9 ± 9.4 cm^2^ at baseline to 2.9 ± 6.6 cm^2^ at follow-up, and 3 (50.0%) limbs improved to C_5_.

## Discussion

Outcomes were decidedly positive in this analysis of patients with IST treated using a newly available mechanical thrombectomy catheter (Central Illustration). Symptom duration ranged from acute to chronic, and almost all limbs were CEAP C_3_ to C_6_ at baseline, indicating that mechanical thrombectomy was viewed across the centers as an appropriate strategy to address a spectrum of IST presentations. The primary effectiveness end point was reached in 100% of limbs even though the obstructive material was classified as chronic on postthrombectomy inspection in 85% of cases, indicating that the study device can reliably debulk obstructive IST irrespective of thrombus chronicity. These results were achieved without stent migrations. Thus, it appears the intervention does not compromise stent function. Also, the proportion of stents relined due to residual thrombus was <10%, suggesting that the thrombectomy device typically provides satisfactory thrombus removal and obviates the need to place an additional venous stent. The results were also durable, with primary patency sustained in 94% of limbs through a median first follow-up of just over 1 month. A study of chronic deep venous disease showed a trend toward superior VEINES-QoL scores at follow-up in patients with sustained primary stent patency (59.7% vs 44.0%; *P* = .06).^21^

**Central Illustration:**
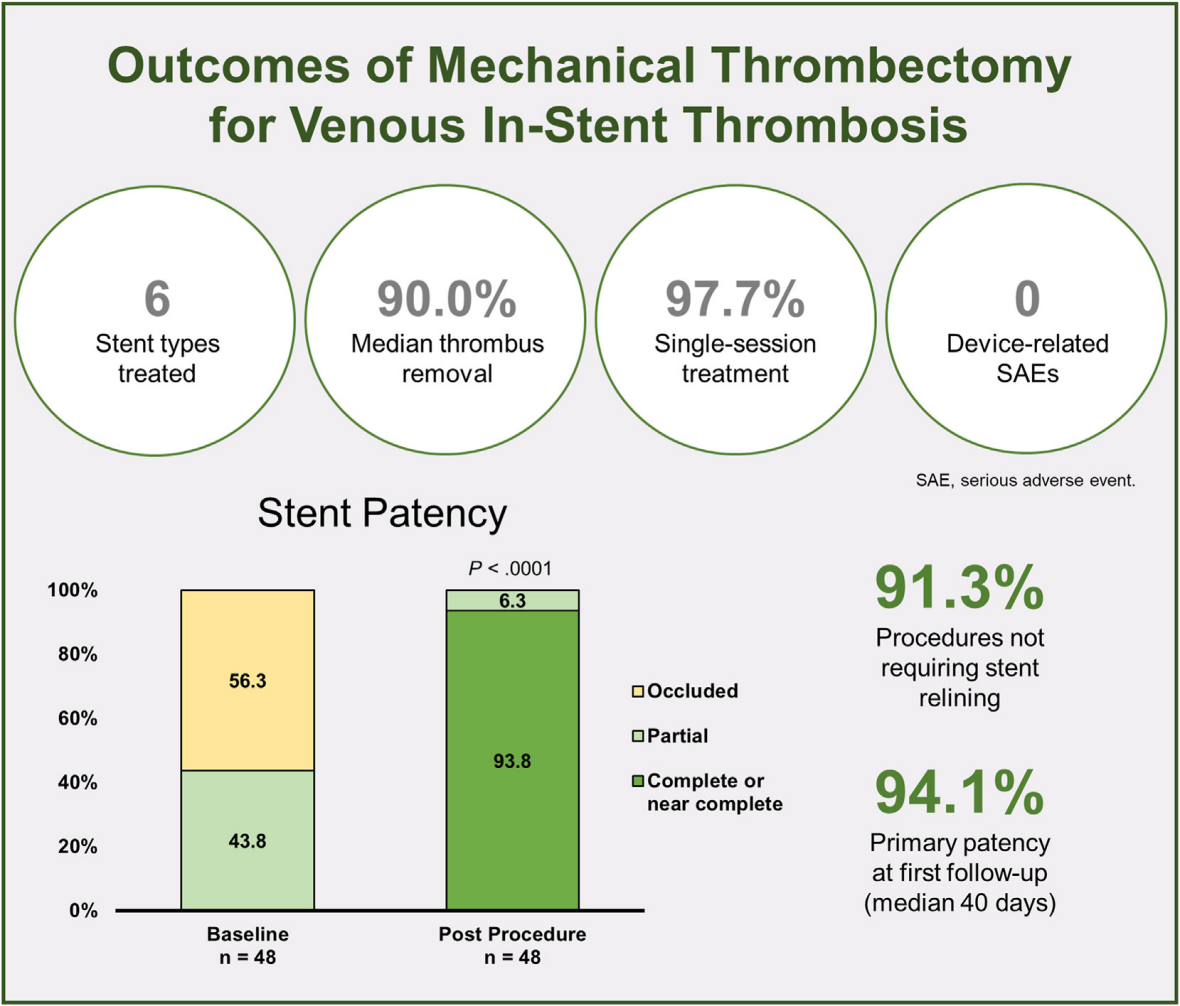
**Key outcomes from a multicenter study of mechanical thrombectomy for venousin-stentthrombosis.** Complete or near-complete stent patency is defined as ≥75% patent; partial stent patency is defined as <75% patent without occlusion.

Importantly, no device-related SAE were identified through 30 days, including no mortalities, readmissions, PE, or vessel perforations. A postprocedural stent fracture was observed, and the experienced operator considered the fracture to be a result of adjunctive prethrombectomy balloon venoplasty. The study device does not possess a built-in means of embolic protection, and procedural embolus was captured in 84% of the cases, highlighting the importance of using a secondary device in the IVC to trap potential emboli. These results suggest that, with appropriate measures to entrap embolized thrombus, this new thrombectomy catheter can be used safely in the setting of IST.

Adverse events occurred during or after 4 (8.7%) of the 46 procedures, and half of these events appeared to be exacerbations of baseline conditions. Interventional treatment is never without risk, and clinicians should discuss the potential benefits and risks of this therapy with the patient, particularly if comorbidities exist. The average patient in this study presented with a Villalta score of 9, corresponding to the high end of the range for mild postthrombotic syndrome on the Villalta scale. Based on the positive outcomes at early follow-up in the present study, the risk-benefit calculation is favorable for patients with significant IST-related symptoms.

The type of IST intervention employed is often guided by the chronicity of obstructive material. Previous research found 69% of iliocaval venous stent occlusions to be chronic on duplex ultrasound,^22^ slightly lower than the 85% of stents observed to include chronic obstructions on postthrombectomy inspection in the present study. For chronic IST, conventional balloon venoplasty is the most common treatment.^22^ One retrospective analysis of venoplasty alone showed that, after dilatation, 62% of stents demonstrated complete clearance of in-stent restenosis (ISR);^12^ the term ISR captures a broader range of reasons for patency loss in addition to thrombosis, such as external compression or neointimal hyperplasia. However, another study of 274 limbs with ISR by Raju et al^13^ showed only a 31% to 42% improvement in flow channel area after venoplasty alone. In the current analysis, outcomes were favorable irrespective of symptom duration or thrombus organization, suggesting that disease chronicity may be of less concern when using this device. Still, both limbs that demonstrated stent occlusion before the first follow-up visit had chronic thrombus removed during the index procedure, suggesting that sustaining stent patency for patients with chronic IST may be challenging and require closer follow-up even in the context of thrombectomy.

Although acute IST is less common than chronic forms of the disease, additional fibrinolytic-based therapies are available in such cases. Pharmacomechanical catheter-directed thrombolysis was the most commonly used approach for acute IST in 1 report, but treatment-specific data were not included.^22^ Ultrasound-assisted thrombolysis has also been used for venous IST. However, a retrospective assessment of 18 patients receiving ultrasound-assisted thrombolysis for either acute or postthrombotic obstructions demonstrated only 61% successful recanalization.^14^ Moreover, adjunctive therapies were used in 73% of cases in that study, and the rate of major bleeds requiring transfusion was 11%, raising concerns about the practicality and safety of thrombolysis for treating patients with venous IST. Of the treated limbs in the current study, 24% were reported to have acute symptoms, but only 3% of the procedures removed exclusively acute thrombus, with the remainder of the procedures involving the removal of more chronic thrombus. This is consistent with the finding in other studies that venous thrombus is often more chronic than symptoms would suggest.^19^^,^^23^ The mismatch in symptom duration versus thrombus chronicity may explain the often poor outcomes achieved with thrombolytic treatments.

The need for improved IST therapies is further highlighted by the large number of patients who develop the condition. Some studies, including a systematic literature review, report loss of stent patency at a rate of roughly 20% to 25% at 2 to 3 years after placement.^7^^,^^8^ Still, other studies have found the rate of IST to be only 3%.^5^^,^^22^ The wide range in occurrence rates may be due to the increased risk of IST in patients with stents placed for thrombotic versus nonthrombotic disease.^9^^,^^10^ Results presented here support this hypothesis, as there was a history of DVT in 96% of the treated limbs. Similarly, patients receiving stents for postthrombotic venous lesions might be more prone to thrombosis when compared with patients receiving a stent for acute DVT.^8^ Even if the proportion of patients who experience IST is only 3%, this equates to a large and growing population with stent obstructions and recurrent venous disease symptoms.

The thrombectomy catheter studied here is the only device specifically designed to treat venous IST and does so by extracting thrombus as opposed to using balloons, stents, or other means to recanalize a vessel via thrombus redistribution or dissolution. In addition, the purely mechanical nature of the therapy and its apparent effectiveness for variably aged thrombus might increase the number of patients with IST who receive interventional care and alter the treatment paradigm. Nevertheless, technical challenges in treating IST remain. For instance, the ability to perform mechanical thrombectomy for IST is dependent on first successfully crossing the lesion, which can present challenges even with contemporary tools.

This analysis is limited by its retrospective design. Prospective studies, like the recently announced REVIT registry (NCT06394739) of patients with IST treated using the study device, will be needed to validate these findings. To better elucidate the common mechanisms of IST occurrence, future research should additionally report both the proportion of IST events that occurred while patients were on anticoagulants and the suspected underlying causes of stent thrombosis. In addition, final stent patency measurements were acquired after any adjunctive therapies, precluding assessment of luminal gain that can be attributed to mechanical thrombectomy alone. The study also lacked specified follow-up windows, and follow-up data were unavailable for some of the patients. Thus, error may have been introduced from follow-up bias. This analysis aimed to describe the outcomes achieved for venous IST using a new mechanical thrombectomy catheter and lacks a comparator arm; future studies should compare the results of thrombectomy versus the current standard of care.

## Conclusion

Results from this multicenter retrospective experience show that the uniquely designed RevCore catheter can successfully treat IST and reestablish stent patency. No device-related SAE occurred, and complete patency was achieved and maintained through follow-up for most stents, demonstrating a favorable safety and effectiveness profile. Additional results, including findings from prospective studies, will be needed to confirm the outcomes obtained here.
